# Antecedents and Consequences of Health Literacy among Refugees and Migrants during the First Two Years of COVID-19: A Scoping Review

**DOI:** 10.3390/tropicalmed9050116

**Published:** 2024-05-16

**Authors:** Kathleen Markey, Uchizi Msowoya, Nino Burduladze, Jon Salsberg, Anne MacFarlane, Liz Dore, Meghan Gilfoyle

**Affiliations:** 1 Department of Nursing and Midwifery and Health Research Institute, University of Limerick, V94 T9PX Limerick, Ireland; 2 School of Medicine, University of Limerick, V94 T9PX Limerick, Ireland; 3 School of Medicine and Health Research Institute, University of Limerick, V94 T9PX Limerick, Ireland; 4 Glucksman Library, University of Limerick, V94 T9PX Limerick, Ireland; 5 Women’s College Hospital Institute for Health System Solutions and Virtual Care, Toronto, ON M5S 1B2, Canada

**Keywords:** refugees and migrants, health literacy, health information, COVID-19, social and environmental determinants, situational determinants, personal determinants

## Abstract

Supporting refugee and migrant health has become a critical focus of healthcare policy. Developing and designing health literacy interventions that meet the needs of refugees and migrants is core to achieving this objective. This literature review sought to identify antecedents and consequences of health literacy among refugees and migrants during the first two years of the COVID-19 pandemic. We systematically searched nine electronic databases and numerous grey literature sources to identify studies published between December 2019 and March 2022. The antecedents (societal and environmental determinants, situational determinants, and personal determinants) and consequences of health literacy among refugees and migrants were mapped to a validated integrated health literacy model. Social and environmental determinants (n = 35) were the most reported antecedent influencing health literacy among refugees and migrants during the first two years of COVID-19. Language (n = 26) and culture (n = 16) were these determinants’ most frequently reported aspects. Situational determinants (n = 24) and personal determinants (n = 26) were less frequently identified factors influencing health literacy among refugees and migrants. Literacy (n = 11) and socioeconomic status (n = 8) were the most frequently reported aspects of personal determinants. Media use (n = 9) and family and peer influence (n = 7) were the most cited situational determinants reported. Refugees and migrants with higher levels of health literacy were more likely to use healthcare services, resulting in better health outcomes. The findings of this review reveal personal and situational factors that impacted health literacy among refugees and migrants during COVID-19 that require attention. However, the inadequate adaptation of health literacy interventions for linguistic and cultural diversity was a greater problem. Attention to this well-known aspect of public health preparedness and tailoring health literacy interventions to the needs of refugees and migrants during pandemics and other public health emergencies are paramount.

## 1. Introduction

Global migration trends have rapidly evolved over the past 20 years. In 2020, there were an estimated 281 million international migrants around the world, which is an increase of 108 million since 2000 [1]. There were also an estimated 26.4 million refugees, an increase of 12.4 million since 2000 [1]. For this study, we refer to the definition of migrant as outlined by the International Organization of Migration [2] and the definition of refugee as outlined by the United Nations High Commissioner for Refugees [3] (Table 1). In an era of expanding global migration trends, providing equitable, accessible, and responsive healthcare for refugees and migrants has become a critical focus of healthcare policy [4]. Nonetheless, reports of health inequalities and disparities in health outcomes for refugees and migrants continue [5,6,7]. Health literacy is increasingly recognized as a fundamental health promotion intervention that can help address such inequalities, as it supports knowledge of health, healthcare systems, and the processing of health information [8]. The Centers for Disease Control and Prevention (CDC) highlights the importance of developing inclusive health communication and this requires paying close attention to the cultural, linguistic, environmental, and historical situation of the intended audience (https://www.cdc.gov/healthliteracy/index.html (accessed on 5 February 2024)). 

The concept of health literacy has evolved since it was first proposed by Simonds [9] as an ability to understand health information related to healthcare. More recently, there have been calls for approaches to health literacy that are relevant to modern society and stretch beyond the view that providing health information in isolation will automatically result in increased health decision-making competencies [8,10]. In 2012, Sørensen and colleagues [11] reported on a systematic literature review of existing definitions and concepts of health literacy, which informed their definition of health literacy and the development of an integrated model of health literacy. In this context, health literacy is defined as ‘knowledge, motivation, and competencies to access, understand, appraise, and apply health information in order to make judgments and take decisions in everyday life concerning healthcare, disease prevention, and health promotion to maintain or improve quality of life during the life course’ [11]. The integrated model outlines the main dimensions of health literacy, antecedents influencing health literacy, and consequences of health literacy [11]. Antecedents of health literacy in this context refer to the multitude of societal and environmental determinants, personal determinants, and situational determinants that influence an individual’s ability to access, understand, appraise, and apply health information (Table 2). Consequences of health literacy are conceptualized as the impact of accessing, understanding, appraising, and applying health information on health service use and subsequent health costs on society, health behavior, and health outcomes, participation/empowerment, and equity/sustainability [11]. 

We know health outcomes, health behavior, and access to healthcare services are improved among refugee and migrant populations when the design and delivery of health literacy interventions are responsive to their needs [12,13]. Despite the growing evidence demonstrating the value of health literacy on health outcomes, reports of inadequate levels of health literacy among refugee and migrant populations persist [14,15,16]. Collectively, this evidence signals the need for developing greater insights into the multitude of factors that influence health literacy among refugee and migrant populations, to optimize future interventions. Further, the COVID-19 pandemic illuminated the necessity of thinking innovatively about the provision of easily accessible and evidence-informed COVID-19-specific and general health information to the whole population, including refugees and migrants, particularly during a public health emergency [17,18,19,20,21,22]. A recent World Health Organization report [23] presented numerous exemplars of refugee and migrant-responsive health literacy interventions developed in response to COVID-19. A systematic review [17] underscored the need for research examining the impact of both general and pandemic-specific health literacy during COVID-19. The pre-COVID-19 evidence about health literacy draws attention to the lack of focused attention to, and at times deficits in, health literacy interventions specifically targeting the needs of refugees and migrants [15,24]. While reviews revealed higher risks of COVID-19 infection rates [25] and increased healthcare access difficulties for refugees and migrants during COVID-19 [26], no reviews have examined factors that enable and hinder health literacy specifically among refugees and migrants during COVID-19. Further, previous literature reviews have not systematically mapped evidence influencing health literacies among refugees and migrants to a comprehensive integrated health literacy model or framework, creating a gap in this area. Arguably, such knowledge could have been used during COVID-19 to optimize pandemic-specific health literacy interventions responsive to the needs of individuals with varying health literacy requirements. Thus, to better understand health literacy influencing factors among refugees and migrants, this review mapped the literature on antecedents and consequences of health literacy published during the first two years of COVID-19 to an evidence-informed conceptual framework of health literacy [11]. 

## 2. Materials and Methods

This review was conducted following the guidance of Peters and colleagues [27], which is an enhancement in previous scoping review guidelines [28,29]. This research arose from an academic–community partnership between the Participatory Health Research Unit, School of Medicine, University of Limerick, and a non-governmental organization (NGO) for migrants in Ireland, Doras. The community partner suggested a participatory prioritization exercise, where a range of stakeholders including migrants, academics, researchers, and healthcare providers identified research priorities for refugee and migrant health (https://www.irishworldacademy.ie/part-im/ (accessed on 1 February 2024)). The importance of knowing how COVID-19 impacted refugees and migrants’ health was named in this prioritization exercise as an area requiring further investigation. The protocol for this scoping review is registered on Open Science Framework (Registration DOI: https://doi.org/10.17605/OSF.IO/HTXD4 (accessed on 3 March 2023)). The Preferred Reporting Items for Systematic Reviews and Meta-Analyses Extension for Scoping Reviews (PRISMA-ScR) [30] was used in the reporting of this review. The PRISMA-ScR checklist can be found in Supplementary File S1. 

The research question for this review incorporates the ‘Population Concept Context (PCC)’ framework, as recommended by Peters and colleagues [27]. Specifically, the population of interest is refugees and migrants, the main concept is health literacy, and the context is the first two years of COVID-19. This led to the formulation of the following review question: What are the antecedents and consequences of health literacy among refugees’ and migrants’ during the first two years of COVID-19? The objectives of this literature review are to:

1. Determine the geographical landscape of the emerging international literature about health literacy for refugees and migrants during the first two years of COVID-19.
2. Identify factors that influence refugees’ and migrants’ abilities to access, understand, appraise, and apply health-related information on healthcare, disease prevention, and health promotion during the first two years of COVID-19.
3. Identify the consequences of health literacy for refugees and migrants during the first two years of COVID-19.

### 2.1. Search Strategy

Using the PCC framework, search terms using keywords and synonyms pertaining to health literacy, migrant or refugee, and COVID-19 were developed. A preliminary search was conducted in Medline, searching titles, abstracts, keywords, and MeSH terms. This preliminary search guided the development of the search strategy used, which was adapted and operationalized across databases, in collaboration with a research librarian (co-author LD). An example search strategy can be found in Supplementary File S2. 

Nine databases were systematically searched: PubMed, CINAHL (EBSCOhost), Scopus (EBSCOhost), Embase (EBSCOhost), MEDLINE-OVID, MEDLINE-FULL TEXT, PsycINFO, Cochrane Library, and Web of Science. In line with recommendations by Godin and colleagues [31], three specific grey literatures sources: Google scholar, Open Grey, and Grey Literature Report; and three key websites: International Organization of Migration, World Health Organization, and United Nations High Commissioner for Refugees, were also searched. Considering the fast pace at which evidence on the impact of COVID-19 is reported, two pre-print sources were also searched. 

### 2.2. Evidence Screening and Selection

Following removal of duplicates in EndNote 20 (n = 1300), the remaining literature was imported to an online screening software package (https://www.rayyan.ai/, accessed on 30 March 2022). A pilot of the eligibility criteria was then completed by the review team (UM, NB, and KM). This involved all three reviewers independently screening 10% of the evidence and then discussing the process and decision-making, and reviewing the eligibility criteria. Once the agreement rate reached 75%, screening commenced using the finalized eligibility criteria (Table 3). The screening was completed independently by two reviewers (UM and NB), firstly by title and abstract (n = 1644) and then by full text (n = 126). All conflicts were discussed and resolved during meetings with the review team (KM, UM, NB). Evidence was included in the review if it met all the eligibility criteria (Table 3). 

### 2.3. Data Charting, Mapping and Synthesis 

A tabular data-charting document was developed and piloted (KM, AM) to determine which variables to extract, in ensuring the review aims and objectives are achieved. General data (e.g., title, authors, year, country), methodological data (e.g., research design, data collection, data analysis, setting), and population characteristics (e.g., sample, size), were extracted. Data specific to factors influencing health literacy and the consequences of health literacy were extracted and mapped to the antecedents and consequences of health literacy as classified in the integrated model of health literacy [11] (see Supplementary Files S3–S5). This model was chosen as a means of exploring health literacy through a conceptual framework lens, whilst specifically mapping the antecedents and consequences of health literacy among refugees and migrants. The charted data were then analyzed and synthesized.

## 3. Results

The PRISMA-ScR flow diagram [30] below illustrates the evidence selection process and provides reasons for exclusion (Supplementary File S6). The searches generated a total of 2944 sources of evidence. Following removal of duplicates, the remaining 1644 sources of evidence were screened by title and abstract. Of the 126 screened at full-text, 47 sources met our inclusion criteria and were included in this review. The results were organized to present a descriptive understanding of the characteristics of included evidence and then to identify the antecedents (societal and environmental, personal, and situational determinants) and consequences of health literacy. 

### 3.1. Overview of Characteristics of Included Evidence

The study characteristics for the 47 extracted sources are presented in Table 4 below. For readability, references are not included in the overview description of extracted sources. 

### 3.2. Antecedents of Health Literacy among Refugees and Migrants during the First Two Years of COVID-19

Of the 47 included sources, 43 identified one or more of the three antecedents as identified by Sørensen and colleagues [11]. Four sources of evidence did not identify antecedents and focused solely on consequences of health literacy [32,33,34,35]. Societal and environmental determinants (n = 35) were the most reported antecedent influencing health literacy among refugees and migrants during the first two years of COVID-19. Situational determinants (n = 24) and personal determinants (n = 26) were less frequently identified factors influencing health literacy among refugees and migrants.

#### 3.2.1. Societal and Environmental Determinants during the First Two Years of COVID-19

Supplementary File S3 summarizes the evidence that shows how societal and environmental determinants affected refugees’ and migrants’ ability to access health information during the first two years of COVID-19. Language (n = 26) and culture (n = 16) were the most frequently reported aspects of these determinants. Accessing health information during COVID-19 was repeatedly reported as a key difficulty for refugees and migrants when there were language barriers [36,37,38,39,40,41,42,43,44,45]. Some sources identified a lack of readily available and translated COVID-19 public health information, creating barriers for refugees and migrants to understand and comply with public health guidelines [40,46,47,48]. Comparatively, health information available in a language that was understood by refugees and migrants resulted in a greater likelihood of accessing timely COVID-19 information [49,50,51]. 

Cultural norms, values, and contexts were identified as key factors influencing refugees’ and migrants’ behaviors and attitudes towards accessing, understanding, appraising, and applying health information [39,41,42,43,47,52,53,54,55,56,57,58,59,60]. The importance of acknowledging and being sensitive to differences in cultural values informing COVID-19 preventative practices [52,54] and general health information [39,60] was also acknowledged. However, a lack of attention to diverse cultural beliefs in the design of COVID-19 health-related information was also reported, creating barriers for access and comprehension of the information [42,47,56,57,61]. Political forces (n = 7), demographic situation (n = 3), and societal systems (n = 2) were the least reported societal and environmental determinants identified. A lack of confidence among refugees and migrants in government responses to COVID-19 outbreaks [43,62], as well as a general mistrust in such sources of COVID-19 information [46,56,63], were the key political forces identified. The impact of inconsistent, infrequent, and changing COVID-19 public health messaging was also reported [56,64].

#### 3.2.2. Personal Determinants during the First Two Years of COVID-19

Supplementary File S4 provides an overview of how personal determinants influenced refugees and migrants’ ability to access health information during COVID-19. Literacy (n = 11) and socioeconomic status (n = 8) were the most frequently reported aspects of personal determinants. Literacy, in terms of the ability to read and understand information, was reported to be a crucial factor in accessing and understanding health information [40,41,50,56,58,65]. The importance of digital literacy as a means of accessing technological health information was also acknowledged [57]. Refugees and migrants with poor literacy relied on family and friends to provide COVID-19 health information, which was less timely or inaccurate [50]. Lower levels of literacy among refugees and migrants resulted in misconceptions about COVID-19 [36] and poor patient outcomes for those living with chronic illness [23]. Refugees and migrants were particularly worried about the impact of COVID-19 on their well-being [50] and were more negatively impacted by ‘lockdowns’, than the general population [66]. Lack of technology and internet access also negatively impacted the abilities to access health information [45,55,56].

Age (n = 5), employment (n = 5), income (n = 4), education (n = 3), and gender (n = 2), were the least reported personal determinants. None of the sources reported race or occupation as influencing factors. Older refugees and migrants had limitations with using technology and subsequently experienced difficulties when accessing digital health information [56,67]. In particular, refugees and migrants in their middle ages were interested in learning about COVID-19 [65,68]. Refugees and migrants with limited paid working opportunities were more likely to access prompt COVID-19 public health information [49]. Refugees and migrants living in low-income households experienced difficulties in accessing health information during the first two years of COVID-19 [51,67,69]. However, those with higher education levels had a better understanding of COVID-19 and preventative practices [47,50,68,70].

#### 3.2.3. Situational Determinants during the First Two Years of COVID-19

Supplementary File S5 provides an overview of how situational determinants influenced health literacy among refugees and migrants during the first two years of COVID-19. Media use (n = 9) and family and peer influence (n = 7) were the most cited situational determinants reported. Social media was identified as the most common source of timely and easily accessible COVID-19-related health information [39,40,42,45,48,65,70]. However, Feinberg and colleagues [58] discussed the challenges of COVID-19 misinformation, which can be rapidly disseminated via social media. Refugees and migrants relied on family members, friends, work colleagues, and/or religious leaders as sources of health information [45,50,51,56,70,71]. Social support (n = 3) and physical environment (n = 1) were frequently described situational determinants. Connecting with social networks was reported as a health literacy support during COVID-19 [36,45,51,71]. Confinement measures due to imposed COVID-19 public health restrictions were physical environment issues that impacted health literacy [57].

### 3.3. Consequences of Health Literacy for Refugees and Migrants during the First Two Years of COVID-19

Table 5 depicts the literature that discussed the consequences of health literacy among refugees and migrants during the first two years of COVID-19. Refugees and migrants who engaged with health information and participated in their healthcare decisions, despite the imposed COVID-19 public health restrictions, were more satisfied with the care received and were more likely to have better health outcomes [33,35,50,67,72]. Refugees and migrants with increased access to health information engaged in more individual or community health programs, adopted healthier behaviors, and generally managed their health more effectively [44,68,70,71,73]. Although refugees and migrants in the middle age group were more likely to have a better understanding of COVID-19 [65], they were less likely to apply COVID-19 preventative practices [68].

Individuals with higher health literacy had better health outcomes during the first two years of COVID-19, including improved health status [44,45,70]. Refugees and migrants with lower socio-economic status were at a higher risk during COVID-19 of developing conditions that are distinctive to poor social determinants of health [36,44,58]. Refugees’ and migrants’ access to and engagement with healthcare services during COVID-19 were influenced by health literacy. Authors [35,44,53,56,66,70,73] suggested that refugees and migrants with higher health literacy were more likely to use healthcare services more effectively, which also resulted in better health outcomes. 

## 4. Discussion

This review adds new insights into the multitude of co-existing factors that influenced health literacy among refugees and migrants at a time when there were imposed public health restrictions and reconfiguration of healthcare services in response to COVID-19. The findings illuminate how language and culture were the most reported aspects of social and environmental determinants that influenced health literacy during the first two years of COVID-19. Most of the evidence identified language barriers and lack of attention to cultural factors influencing perceptions of general and COVID-19-specific health information as key obstacles to accessing, understanding, appraising, and applying health information. The poignant impact of the lack of culturally appropriate approaches to the design and development of health information and inconsistent implementation of effective cross-cultural health information communication strategies is a key finding of this study. The findings also identify how media, family, and peer influences, as situational determinants, had a key role in influencing health literacy among refugees and migrants during the first two years of COVID-19. For example, the findings indicate a general mistrust among refugees and migrants in COVID-19 public health information distributed by governments. Consequently, refugees and migrants were more likely to turn to unofficial sources of information such as media, family, peers, and community networks. Refugees and migrants with greater ability to access, understand, appraise, and apply health information were more empowered to make informed healthcare decisions and were more likely to take appropriate actions to manage their own health. However, there were also negative health outcomes reported due to difficulties in accessing or understanding health information, such as increased COVID-19 infection rates, poorer outcomes for those living with chronic illness, and lack of adherence to COVID-19 public health guidelines.

Thus, although the range of health literacy interventions identified in this review targeting the needs of refugees and migrants in response to COVID-19 is welcomed, the findings of this study highlight what is already well known [4]. Health care systems and health education interventions need adaptation for cultural and linguistic diversity in order to eliminate systemic barriers for refugees and migrants in accessing healthcare systems [7,74,75]. This is an important message from this review because the emphasis in the field of health literacy is not always in line with this. Certainly, the findings of this review reveal the importance of designing health literacy interventions for refugees and migrants who, for example, may have varied levels of general literacy. However, Berens and colleagues [76], assert the importance of avoiding making stereotypical assumptions about the general literacy levels of refugees and migrants in the planning of health literacy interventions. Similarly, the benefits associated with the use of health information technology among general populations during COVID-19 are acknowledged [77]. However, this review found that there were some difficulties reported in terms of availability and ability to use technology, and for some there was limited access to networks, which negatively impacted access to health information. This means that it is important to avoid assumptions about digital competencies or resources. 

Moreover, as mentioned, the findings of this review emphasize the critical roles that language and culture play in influencing health literacy among refugee and migrant populations, echoing findings reported elsewhere [6,78,79]. Although others have also highlighted the importance of acknowledging the interconnections of health literacy, language, and culture [80], it is an area that has received limited attention to date within health literacy literature. The findings of this study illustrate the importance of paying close attention to language and culture when designing and implementing health literacy interventions as a means of meeting the needs of refugees and migrants, mirroring recommendations identified by others [81]. 

The National Action Plan to Improve Health Literacy as developed by the U.S. Department of Health and Human Services [82] provides strategic direction when developing culturally and linguistically appropriate health literacy interventions as a means of improving health literacy for all communities. This study highlights the importance of developing participatory, community-based peer support approaches to designing and disseminating inclusive health literacy interventions. These recommendations align with the recent CDC (Centers for Disease Control and Prevention) guiding principles to promoting an equity-centered approach to health literacy, and recommendations in the WHO European region [83,84]. Collaboratively engaging with refugee and migrant communities in co-creating targeted health literacy interventions ensures the cultural and linguistic appropriateness of the intervention. Using participatory approaches in co-designing health literacy interventions increases health literacy effectiveness and sustainability [85]. However, this requires a collective commitment to grow integrated inter-agency and cross-sector working as a means of strengthening community-based peer support approaches. 

## 5. Methodological Critique

The co-design of the research question with refugees and migrants (https://www.irishworldacademy.ie/part-im/ (accessed on 1 February 2024)), which is in line with recommendations for the field [84], is a key strength of this study. Other strengths include the use of the updated scoping review method [27], collaborating with a librarian, and incorporating a health literacy conceptual framework [11].

There are limitations that must also be acknowledged. Firstly, the evidence presented in this review includes literature published between January 2020 and March 2022, as we wanted to examine literature published during the first two years of COVID-19. The literature published prior to this and other key evidence that became available following March 2022 may highlight additional evidence. However, the key findings resonate with the wider literature. Further, our use of Sorensen’s framework [11] adds some conceptual depth to the analysis of included literature. Secondly, the limited, unclear, and interchangeable use of refugee and migrant definitions across sources of evidence, and sometimes even within the individual source of evidence, meant it was not possible to map this data across evidence. This is an issue that requires attention in the field more broadly [84,86]. Finally, data about country of origin, length of stay in the host country, and residency type were not extracted, and these would be worth close examination in future studies, given that these factors do impact health literacy among refugees and migrants.

## 6. Conclusions

This review re-affirms the complexities of planning health literacy-related interventions and adds to this evidence by illuminating these complexities among refugees and migrants within the context of the first two years of COVID-19. The inadequate adaptation of health literacy interventions for linguistic and cultural diversity was a particular problem that requires attention. Strategically planning tailored culturally and linguistically appropriate health literacy interventions during pandemics and other public health emergencies is paramount.

## Figures and Tables

**Table 1:** Definitions of refugee and migrant.

Refugee	A refugee is defined as ‘a person who owing to a well-founded fear of being persecuted for reasons of race, religion, nationality, membership of a particular social group, or political opinion, is outside the country of their nationality and is unable to or, owing to such fear, is unwilling to avail himself/herself of the protection of that country’ [3].
Migrant	A migrant is defined as ‘any person who is moving or has moved across an international border or within a state away from his/her habitual place of residence, regardless of the person’s legal status, whether the movement is voluntary or involuntary, what the causes for the movement are and what the length of the stay is’ [2].

**Table 2:** Classification of antecedents of health literacy [11].

Sørensen et al. (2012) [11] classify the antecedents of health literacy as; societal and environmental determinants, situational determinants, and personal determinants, which influence the ability to access, understand, appraise, and apply health information on healthcare, disease prevention and health promotion. Societal and environmental determinants include; demographic situation, culture, language, political forces and societal systems [11]. Personal determinants include; age, gender, socioeconomic status, education, employment, literacy, race and occupation [11]. Situational determinants include; physical environment, media use, family and peer influence and social support [11].

**Table 3:** Eligibility criteria.

Criterion	Inclusion	Exclusion	Rationale
Population—Refugee and migrants	Focus on refugee and migrants of all ages	Populations that are not considered as refugees or migrants (as defined in background) such as internal migrants, host populations, and Indigenous populations Populations without any representation of refugees or migrants	To specifically explore health literacy among refugees and migrants
Concept—health literacy	Evidence that discusses health literacy (access, understanding, appraising, applying health information)	Evidence that does not discuss health literacy (access, understanding, appraising, applying health information)	To describe the extent of existing evidence on health literacy among refugee and migrants
Context—the first two years of COVID-19	Evidence that specifically focuses on the first two years of COVID-19 Evidence published between December 2019 and March 2022	Evidence that focuses on epidemics, endemics and other pandemics Evidence published before December 2019 or after March 2022	The novel nature of COVID-19 and the impact of COVID-19 public health restrictions which required varied adaptations of approaches to the provision of accessible and evidence-informed COVID-19-specific and general health information, particularly during the early stages of COVID-19 COVID-19 was first identified in December 2019, but was declared a pandemic in March 2020
Type of Literature	Peer reviewed empirical studies, pre-print literature and grey literature (to specifically include theses/dissertations, policy briefs, reports, conference proceedings, editorials, commentaries, opinion pieces, and discussion papers)		Capture a comprehensive body of evidence
Language	Published in English	Published in any language other than English	Lack of resources and the review team only speak English
Study location	All geographical locations—an international context	None	Exploring perspectives from a global context

**Table 4:** Overview description of extracted sources.

Variable	Total-n (%)
Total-n	47
- Geographical location of literature	
- Africa: Kenya (n = 1)	1 (2%)
- Asia: Bangladesh (n = 1), Mainland of China (n = 3), Hong Kong, China (n = 2), Japan (n = 1), Singapore (n = 2), South Korea (n = 1), Thailand (n = 1)	11 (23%)
- Australia (n = 1)	1 (2%)
- Europe: Austria (n = 1), Denmark (n = 1), France (n = 1), Germany (n = 1), Italy (n = 1), Norway (n = 3), Portugal (n = 1), Spain (n = 2), Sweden (n = 1), United Kingdom (n = 8)	20 (43%)
- Middle East: Israel (n = 3), Jordan (n = 2), Turkey (n = 1)	6 (13%)
- North America: USA (n = 8)	8 (17%)
Date published	
- 2020 - 2021 - Jan–March 2022	12 (26%) 26 (55%) 9 (19%)
Publication type	
- Empirical - Non-empirical	30 (64%) 17 (36%)
Empirical study design	
- Qualitative - Quantitative - Mixed methods - Literature review	11 (37%) 10 (33%) 3 (10%) 6 (20%)

**Table 5:** Consequences of health literacy.

Authors (Year)	Consequences of Health Literacy					
	Participation	Engagement	Influences on Health Behavior	The Use of Health Services	Impact on Health Outcomes	Impact on the Costs in Society
Zlotnick et al. (2022) [50]	X					
Zamil et al. (2022) [65]			X			
Wang et al. (2020) [67]		X				
Tan et al. (2021) [73]	X			X		
Pereira et al. (2021) [46]					X	
Papwijitsil et al. (2021) [68]		X	X			
Oktavianus and Lin (2021) [71]		X	X			
Nezafat et al. (2020) [32]			X			
Narla et al. (2020) [44]		X		X	X	
Mistry et al. (2021) [36]			X			
Madar et al. (2022) [33]			X			
Aragona et al. (2020) [66]				X		
Lu and Chu (2022) [53]			X	X		
Knights et al. (2021) [45]			X		X	
Khader et al. (2022) [35]	X			X		
Healey et al. (2022) [56]				X		
Liem et al. (2021) [70]		X		X	X	

## Data Availability

The original contributions presented in the study are included in the article/Supplementary Materials; further inquiries can be directed to the corresponding author.

## Supplementary Materials

The following supporting information can be downloaded at: https://www.mdpi.com/article/10.3390/tropicalmed9050116/s1, File S1: Preferred Reporting Items for Systematic reviews and Meta-Analyses extension for Scoping Reviews (PRISMA-ScR) Checklist; File S2: Search Strategy; File S3: Societal and environmental determinants; File S4: Personal determinants; File S5: Situational determinants; File S6: PRISMA-ScR flow diagram. References [87,88,89] are cited in the supplementary materials.
