# Supplementary material for: Antecedents and Consequences of Health Literacy among Refugees and Migrants during the First Two Years of COVID-19: A Scoping Review
Source: Trop Med Infect Dis. 2024 May 16;9(5):116. doi: 10.3390/tropicalmed9050116 (PMC11126087; doi:10.3390/tropicalmed9050116)
Supplement: Supplementary file 1 [file tropicalmed-09-00116-s001.zip › Supplementary File S2 Search Strategy.pdf]

**Supplementary File S2 – Search Strategy**

| Search | Search term |
|---|---|
| #1 | (MM "Health Literacy") OR (MM "Health Education") OR "health communication" OR (MM "Health Behavior") OR (MH "Consumer Health Information") OR (MM "Health Knowledge") |
| #2 | TI ( health n3 ( literacy or education or knowledge or communication or behavi?r or information or competence or promotion) ) OR AB ( health n3 ( literacy or education or knowledge or communication or behavi?r or information or competence or promotion) ) |
| #3 | TI ( e-health literacy or ehealth literacy or consumer health information or medical information ) OR AB ( e-health literacy or ehealth literacy or consumer health information or medical information ) |
| #4 | TI health knowledge, attitudes, practice OR AB health knowledge, attitudes, practice |
| #5 | #1 OR #2 OR #3 OR #4 |
| #6 | TX migrant* or emigrants AND immigrants or immigrant or transients AND migrants or refugee* or asylum* or minority groups or ethnic groups or ethnicity or migrat* or emigrant* or emigrat* or immigrat* |
| #7 | TX nCov-2019 or 2019nCov or 2019ncov or novel coronavirus |
| #8 | TX Covid-19 or covid 19 or SARs-COV-2 or covid19 or coronavirus |
| #9 | #7 OR #8 |
| #10 | #5 AND #6 AND #9 |
