# Supplementary material for: Antecedents and Consequences of Health Literacy among Refugees and Migrants during the First Two Years of COVID-19: A Scoping Review
Source: Trop Med Infect Dis. 2024 May 16;9(5):116. doi: 10.3390/tropicalmed9050116 (PMC11126087; doi:10.3390/tropicalmed9050116)
Supplement: Supplementary file 1 [file tropicalmed-09-00116-s001.zip › Supplementary File S3 societal determinants.pdf]

Supplementary File S3 Societal and Environmental Determinants

Culture	Author/Year	Examples from literature
	Quandt, et al. (2020)	"The inconsistency between women in farmworker families seeing themselves as avoiding situations for infection and their actual practices may be due to their living situations and to cultural values" (Quandt et al., 2020)
	Lu and Chu (2022)	"…we found that acculturation, accuracy and defense motivations, trust in media, and perceived information gathering capacity played a key role in explaining information seeking from an intercultural viewpoint, and that the use of U.S. and Chinese media was associated with different health behaviors" (Lu and Chu, 2022)
	Levin-Zamir, et al. (2021)	"Addressing the needs of vulnerable populations in the appropriate language and cultural context is key"(Levin-Zamir et al., 2021)
	Lee, et al. (2020)	"Even when the infrastructure does exist, online health information that is relevant to farmworkers is diffuse and not always culturally or linguistically sensitive"(Lee et al., 2020)
	Kiyohara, et al. (2022)	"Vulnerable migrants, especially those with culturally and linguistically diverse backgrounds, should not be marginalized regarding information access during a health crisis."(Kiyohara et al., 2022)
	Kananian, et al. (2021)	"The lack of knowledge on COVID-19 in refugees might be explained by language problems and cultural barriers to the access of health information"(Kananian et al., 2021)
	Ho and Smith (2020)	"Given the poor ability of foreign domestic workers to access health-related information from nurses and the language and cultural barriers that they face, a territory-wide assessment of their health literacy, as well as its association with acculturation"(Ho and Smith, 2020)
	Healey, et al. (2022)	"Service providers described how Ezidi socio-cultural norms relating to age and gender influenced the way community members sought and shared COVID-19 messages"(Healey et al., 2022)
	Deal, et al. (2021)	"Structural stigmatisation, social exclusion, and cultural and physical barriers to accessing mental health services may further deteriorate an already fragile population…"(Deal et al., 2021)
	Germain and Yong (2020)	"… by critiquing barriers to accessing healthcare from a feminist standpoint, we show that there are specific barriers for women aggravated by their race and cultural associations with race, or having migrant status and being subject to hostile immigration law"(Germain and Yong, 2020)
	Feinberg, et al. (2021)	"Low levels of literacy and health literacy combined with language and cultural differences are common challenges to delivering health information in vulnerable refugee, immigrant, and migrant communities; however, COVID-19 presented an unprecedented challenge requiring immediate and urgent risk communication"(Feinberg et al., 2021)

	Crawshaw, et al. (2021)	"This may partially explain why more recently arrived and less acculturated migrants were at greater risk of under-vaccination"(Crawshaw et al., 2021)
	Brønholt, et al. (2021)	"This included how and where to find health and risk information, or making sense of the information they found, either because it was in Danish or imbued with cultural reference points"(Brønholt et al., 2021)
	(Bojorquez et al., 2021)	"In LACs, migrants face barriers in accessing regular health services due to inadequate information, the absence of culturally appropriate care, or insufficient legal provisions."(Bojorquez et al., 2021)
	Alemi, et al. (2020)	"Recommending epidemiologic risk assessments and the timely deployment of outbreak response teams within refugee camps, promoting health education in a culturally sensitive manner, and ensuring health care access without refoulment for refugees"(Alemi et al., 2020)
	Al-Oraibi, et al. (2021)	"These populations also experience multiple barriers to public and health services, including discrimination, insecure legal status, restrictive policies, limited knowledge of health systems, linguistic and cultural barriers, and mistrust of authorities…" (Al-Oraibi et al., 2021)
**Language**	**Author/Year**	**Examples from literature**
	Zlotnick, et al. (2021)	"Our study found that migrants who reported having better language abilities in the host country's mother tongue, were more likely to access COVID-19 information on the same day it was issued"(Zlotnick et al., 2021)
	Zlotnick, et al. (2022)	"Under the Process component, respondents reported average language ability, almost two-thirds had health literacy (received accurate information and obtained the information when it was first announced), more than three-quarters reported receiving public health information accurately, and almost two-thirds received the information when it was first announced"(Zlotnick et al., 2022)
	Zamil, et al. (2022)	"These results underscore the importance of adopting language-tailored culturally competent COVID-19 educational programs for MENA immigrants, as customized programs may lead to greater acceptance of COVID-19 vaccinations, higher knowledge, and improved practice of protective health behaviors"(Zamil et al., 2022)
	Wang, et al. (2021)	"Communication barriers due to language may impede direct clinical care. The communication barriers may also generate fear of discrimination which may alter health seeking behavior by these populations"(Wang and Teo, 2021)
	Wang, et al. (2020)	"Barriers to healthcare accessibility, such as low income, poor health literacy and language differences further compounded infection risks"(Wang et al., 2020b)

	Wang, et al. (2020)	"However, our study also showed that many international migrants were dubious about received information regarding COVID-19 and Chinese people. This may be due to cultural differences, a language barrier, or stigma and discrimination"(Wang et al., 2020a)
	Pereira, et al. (2021)	"As we investigated further, we observed that public health information was being deployed most often in English and from a majority-predominant point of view. As a result, minority and immigrant communities were struggling to understand the dangers of COVID-19, trust the medical community, and act on guidelines for social distancing"(Pereira et al., 2021)
	Narla, et al. (2020)	"Multiple barriers to care access exist, including language differences, navigational challenges, lack of medical records, fear of deportation, and xenophobia(Narla et al., 2020)
	Mistry, et al. (2021)	"Language is one of the greatest barriers to access health information among immigrants and refugees…"(Mistry et al., 2021)
	Lu and Chu (2022)	"We employed two types of acculturation measures: a more traditional one based on host country language and friends, and another focused on perceived distance from both host and home countries which might be a better representation of the bidimensional model of acculturation."(Lu and Chu, 2022)
	Levin-Zamir, et al. (2021)	"Addressing the needs of vulnerable populations in the appropriate language and cultural context is key"(Levin-Zamir et al., 2021)
	Lee, et al. (2020)	"Even when the infrastructure does exist, online health information that is relevant to farmworkers is diffuse and not always culturally or linguistically sensitive"(Lee et al., 2020)
	Knights, et al. (2021)	"Language barriers were repeatedly reported by migrants and primary care professionals alike, and were perceived to have increased due to digitalization"(Knights et al., 2021)
	Kiyohara, et al. (2021)	"Vulnerable migrants, especially those with culturally and linguistically diverse backgrounds, should not be marginalized regarding information access during a health crisis"(Kiyohara et al., 2022)
	Kananian, et al. (2021)	"The lack of knowledge on COVID-19 in refugees might be explained by language problems and cultural barriers to the access of health information"(Kananian et al., 2021)
	Jang, et.al. (2021)	"Third, although we found some individual-level barriers to health information, such as inadequate language proficiency and lack of medical insurance, no specific individual-level characteristics (e.g., age, educational level, income, and state of residence) were available"(Jang et al., 2021)
	Ho and Smith (2020)	"The ability of foreign domestic workers to follow these recommendations is severely inhibited when the available information is not provided in their native language shortly after the onset of the pandemic"(Ho and Smith, 2020)

	Harris, et al. (2021)	“The challenges to COVID-19 knowledge and prevention included Karen elders lack of literacy in English or native language; household crowding resulting in limited social distance options when essential workers are traveling between the house and work; and availability of supplies (early pandemic) and cost (mid-pandemic)”(Harris et al., 2021)
	Deal, et al. (2021)	“Taking into account the richly diverse background of cultures, religions, and languages may help increase access to these interventions and promotion programmes…”(Deal et al., 2021)
	Goldsmith, et al. (2022)	“Among migrant and ethnic minority populations in the UK, US, China, Jordan, Qatar, and Turkey we found evidence of consistent use of social media for COVID-19 information, including via WeChat, Facebook, WhatsApp, Instagram, Twitter, YouTube, which may stem from a difficulty in accessing COVID-19 information in their native languages or from sources they trusted”(Goldsmith et al., 2022)
	Germain and Yong (2020)	“We identify three categories: information, language, and communication barriers, gendered cultural norms and stigma associated with seeking care.”(Germain and Yong, 2020)
	Feinberg, et al. (2021)	“Low levels of literacy and health literacy combined with language and cultural differences are common challenges to delivering health information in vulnerable refugee, immigrant, and migrant communities; however, COVID-19 presented an unprecedented challenge requiring immediate and urgent risk communication” (Feinberg et al., 2021)
	Crawshaw, et al. (2021)	“Access barriers were very common in the literature and related to language, literacy and communication barriers, practical and legal barriers to vaccination services and systems, and service barriers (including lack of dedicated resourcing, specific guidelines, and training/knowledge of healthcare professionals) for key vaccines, including MMR, DTP, HPV, influenza, polio, COVID-19 vaccine”(Crawshaw et al., 2021)
	Brønholt, et al. (2021)	“Another common barrier to the migrants’ ability to navigate the pandemic response was linguistic difficulties…”(Brønholt et al., 2021)
	Lusambili, et al. (2021)	“Additional efforts were made to provide simple information using posters as well as translating the information into languages well understood by refugees”(Lusambili et al., 2021)
	Alemi, et al. (2020)	“This situation is compounded by language barriers that refugees face in host communities and their limited access to health care for obtaining health information, testing and treatment, which some may even avoid out of fears of being deported”(Alemi et al., 2020)
	Al-Oraibi, et al. (2021)	“These populations also experience multiple barriers to public and health services, including discrimination, insecure legal status, restrictive policies, limited knowledge of health systems, linguistic and cultural barriers, and mistrust of authorities…”(Al-Oraibi et al., 2021)

Political forces	Author/Year	Examples from literature
	Al-Oraibi, et al. (2021)	"In this study, we described for the first time, confidence in government and the spread of rumours amongst migrant workers involved in COVID-19 outbreaks."(Al-Oraibi et al., 2021)
	Pereira, et al. (2021)	"Recognizing that our immigrant communities are prone to mistrust government authorities, we identified Hispanic and Egyptian community leaders to be the primary voices and faces of our outreach"(Pereira et al., 2021)
	Lupieri (2021)	"Second, national responses have frequently neglected to provide adequate public health information to refugees"(Lupieri, 2021)
	Koval, et al. (2022)	"Regarding risk communication, the issue of trust in sources of information and ways of gaining information from authorities was raised"(Koval et al., 2021)
	Hoefer, et al. (2021)	"Because of resource constraints, health education and risk communication activities in reception centers may not occur as frequently as they did at the beginning of the pandemic."(Hoefer et al., 2021)
	Healey, et al. (2022)	"Moreover, participants felt that inconsistent and frequently changing COVID-19 messages across Australian states had contributed to the community's distrust in governmental information"(Healey et al., 2022)
	Lusambili, et al (2021)	"Political and religious factors, fear of contracting COVID-19 and non-compliance with COVID-19 preventive messages and measures were mentioned as contributing to the spread of the disease…" (Lusambili et al., 2021)
**Societal forces**	**Author/Year**	**Examples from literature**
	Mistry, et al. (2021)	"Furthermore, among Rohingya, social and religious taboos, and orthodox thinking, which is more common..."(Mistry et al., 2021)
	Lusambili, et al. (2021)	"The study data suggest that, predominantly Muslim refugee communities initially believed that COVID-19 could affect only Christians as a punishment from God and that Muslims would be protected if they continued to pray five times a day..."(Lusambili et al., 2021)
**Demographic situation**	**Author/Year**	**Examples from literature**
	Papwijitsil, et al. (2021)	"Our study also found that a longer stay in Thailand was associated with lower awareness about public health measures"(Papwijitsil et al., 2021)
	Lupieri (2021)	"First, the healthcare needs of refugees living in overcrowded conditions in refugee camps and detention centers in both high- and low-income settings have been largely overlooked"(Lupieri, 2021)

	Lee, et al. (2020)	“Telemedicine services require strong Internet connection and audiovisual hardware that, due to geography and economic conditions, are often unavailable to farmworkers…”(Lee et al., 2020)
