# Supplementary material for: Antecedents and Consequences of Health Literacy among Refugees and Migrants during the First Two Years of COVID-19: A Scoping Review
Source: Trop Med Infect Dis. 2024 May 16;9(5):116. doi: 10.3390/tropicalmed9050116 (PMC11126087; doi:10.3390/tropicalmed9050116)
Supplement: Supplementary file 1 [file tropicalmed-09-00116-s001.zip › Supplementary file S4 Personal Determinants.pdf]

Supplementary File S4 – Personal Determinants

	Author/Year	Examples from literature
**Age**	Zlotnick, et al. (2022)	"…however, among them, age, being worried about getting COVID-19 and the interaction term demonstrated stronger effects"(Zlotnick et al., 2022)
	Zamil, et al. (2022)	"…those in the age group 18–25 years old were more likely to have "excellent/good" knowledge on preventing COVID-19 spread compared to the age group of 40 years and above"(Zamil et al., 2022)
	Wang, et al. (2020)	"Telehealth-compatible devices or access to the Internet may be unattainable to low-income households or challenging to adopt by the elderly who are less technologically savvy"(Wang et al., 2020b)
	Papwijitsil, et al. (2021)	"However, being middle-aged (compared with being young) was significantly associated with fewer preventive practices"(Papwijitsil et al., 2021)
	Healey, et al. (2022)	"Older community members stated that they are not familiar with technology; therefore, they relied on younger members to receive COVID-19 messages." (Healey et al., 2022)
**Gender**	Quandt, et al. (2020)	"Together, these findings give a sense that, while the women in farmworker families had somewhat better knowledge, they perceived less personal susceptibility to COVID-19."(Quandt et al., 2020)
	Healey, et al. (2022)	"Additionally, service providers thought that information delivered by women might be less acceptable to men"(Healey et al., 2022)
**Race**	-	-
**Socioeconomic status**	Zlotnick, et al. (2022)	"Such that lower perceived stress was noted among those with good/very good SES, retired or employed, and those who were "not really" worried about getting COVID-19"(Zlotnick et al., 2022)

	Narla, et al. (2020)	“Further, refugees experience many conditions that are characteristic of poor social determinants of health: low socioeconomic status, social exclusion, baseline poor nutrition, and unsafe living conditions “ (Narla et al., 2020)
	Mistry,et al. (2021)	“Among the refugee population where scarcity of safe water and nutritious food is paramount, their perceptions about these commodities’ positive effects are understandable”(Mistry et al., 2021)
	Aragona, et al. (2020)	“As a result of the COVID-19 pandemic and consequences of lockdown, migrants and, more generally, individuals in poor socioeconomic conditions can experience a greater negative impact than the general population”(Aragona et al., 2020)
	Lee, et al. (2020)	“Telemedicine services require strong Internet connection and audiovisual hardware that, due to geography and economic conditions, are often unavailable to farmworkers”(Lee et al., 2020)
	Knights, et al. (2021)	“Although some primary care professionals disagreed, many were concerned that a lack of technology, along with challenges in using it, are barriers to access”(Knights et al., 2021)
	Healey, et al. (2022)	“Access to technology equipment, such as a device or Internet was reported to be problematic”(Healey et al., 2022)
	Gosselin et al., (2021)	“Additionally, it is likely that overcrowded housing and working conditions in frontline jobs have resulted in greater risk of Covid-19 exposure, whereas economic hardship and deteriorated mental health may have decreased individuals’ access to healthcare, even in case of Covid-19 symptoms” (Gosselin et al., 2021)

	Alemi, et al. (2020)	"The economic crisis caused by efforts to contain the pandemic is worsening the refugees' already precarious situation in informal labour markets..."(Alemi et al., 2020)
**Education**	Papwijitsil, et al. (2021)<br><br>Kananian, et al. (2021)<br><br>Liem, et al. (2021)	"Frequent reception of health information and primary school completion showed a statistically significant association with a high level of preventive practices, relative to those who rarely received health information and had no formal schooling…"(Papwijitsil et al., 2021)<br><br>"This impairment may be additionally increased by a lower educational level as reported in our refugee sample…" (Kananian, et al., 2021)<br><br>"Educational level was a key factor associated with virus knowledge, which is similar to survey findings on COVID-19 knowledge among residents in mainland China during the early phase of the COVID-19 pandemic"(Liem et al., 2021)
**Occupation**	-	-
**Employment**	Zlotnick, et al. (2021)<br><br>Feinberg, et al. (2021)<br><br>Elisabeth, et al. (2020)<br><br>Al-Oraibi, et al. (2021)	"Additionally, findings showed that migrants who obtainedCOVID-19 information on the same day it was issued were more likely to be those who had decreased employment or unemployment after COVID-19 government restrictions, contrary to the hypothesis…(Zlotnick et al., 2021)<br><br>"Not only do refugees work in jobs where they may be exposed to COVID-19 (and also often live in overcrowded housing where there is increased risk of transmission), but they are subject to layoffs, reduced work hours, or unemployment"(Feinberg et al., 2021)<br><br>"Refugees in Sweden are already facing challenges such as poor health, difficulties with employment, crowded living conditions and difficulties obtaining health care as well as understanding health care information"(Elisabeth et al., 2020)<br><br>"Migrants experience multiple risk factors for SARS-CoV-2 infection and adverse clinical outcomes, including poor or overcrowded living conditions, employment in informal or essential roles with inability

		to work from home, sparse access to adequate water, sanitation, and hygiene services, and complex health needs including cardiometabolic comorbidities"(Al-Oraibi et al., 2021)
	Liem, et al. (2021)	"In addition, approximately one-third of participants who work as domestic or care workers (e.g., cleaners, cooks, and servers) knew less about drug/vaccine and prevention strategies than their counterparts…"(Liem et al., 2021)
**Income**	Wang, et al. (2020)	"Telehealth-compatible devices or access to the Internet may be unattainable to low-income households or challenging to adopt by the elderly who are less technologically savvy"(Wang et al., 2020b)
	Germain and Yong (2020)	"We note that all three scenarios disproportionately impact those on low incomes, which ultimately affect women more because of the high numbers of women in low-income jobs, especially because of the numbers of single mothers supporting children"(Germain and Yong, 2020)
	Feinberg, et al. (2021)	"In general, these community members live in high-density housing; work frontline jobs in manufacturing, healthcare, and food processing; are under- or uninsured; and lack access to healthcare providers for both culturally and linguistically concordant information and care"(Feinberg et al., 2021)
	Lusambili, et al. (2021)	"The data suggest that refugee family size and the economic challenges they faced might have influenced whether they adhered to COVID-19 measures"(Lusambili et al., 2021)

**Literacy**	Zlotnick, et al. (2022)	"A plausible explanation for this negative association could be that migrants, due to lack of education or understanding of other sources of information, relied on friends and family as their health resource; and reliance on friends and family as their sole health information source resulted in obtaining health information in a less timely or accurate manner"(Zlotnick et al., 2022)
	Zamil, et al. (2022)	"Findings from our study also suggest that individuals who reported relatively adequate knowledge on how to protect themselves from COVID-19 infection also reported higher levels of COVID-19 prevention knowledge"(Zamil et al., 2022)
	Wernly, et al. (2020)	"Lower rates of health literacy and signals towards worse control of chronic diseases in migrants could lead to significantly worse outcomes in this population"(Wernly et al., 2020)
	Mistry, et al. (2021)	"One apparent reason for the prevailing misconceptions about COVID-19 among study participants could simply be due to low literacy; ~98% of participants were illiterate…"(Mistry et al., 2021)
	Lu and Chu (2022)	"By contrast, the language-based measure was potentially more relevant because utilizing U.S. media required a certain level of English proficiency"(Lu and Chu, 2022)
	Kananian, et al. (2021)	"This is in line with findings that show less knowledge and poor health care access for migrants compared to non-migrants"(Kananian et al., 2021)
	Healey, et al. (2022)	"Community members and influential community members reported low literacy, not only in English, but also Kurdish-Kurmanji…"(Healey et al., 2022)
	Harris, et al. (2021)	"However, among the Karen participants, it was shared that readily available information on COVID-19 in Karen across various channels would be helpful for Karen elders because the elders are not all literate in English or their native language"(Harris et al., 2021)

	Deal, et al. (2021)	“Second, we suggest that low digital literacy and reduced access to technological means should be considered a top barrier to overcome when designing tele-mental health services”(Deal et al., 2021)
	Feinberg, et al. (2021)	“Low levels of literacy and health literacy combined with language and cultural differences are common challenges to delivering health information in vulnerable refugee, immigrant, and migrant communities; however, COVID-19 presented an unprecedented challenge requiring immediate and urgent risk communication”(Feinberg et al., 2021)
	Crawshaw, et al. (2021)	“Access barriers were very common in the literature and related to language, literacy and communication barriers, practical and legal barriers to vaccination services and systems, and service barriers (including lack of dedicated resourcing, specific guidelines, and training/knowledge of healthcare professionals) for key vaccines, including MMR, DTP, HPV, influenza, polio, COVID-19 vaccine…”(Crawshaw et al., 2021)
