# Supplementary material for: Antecedents and Consequences of Health Literacy among Refugees and Migrants during the First Two Years of COVID-19: A Scoping Review
Source: Trop Med Infect Dis. 2024 May 16;9(5):116. doi: 10.3390/tropicalmed9050116 (PMC11126087; doi:10.3390/tropicalmed9050116)
Supplement: Supplementary file 1 [file tropicalmed-09-00116-s001.zip › Supplementary File S5 Situational Determinants.pdf]

Supplementary File S5 Situational Determinants

	Author/Year	Examples from literature
**Social support**	Oktavianus and Lin (2021)	"MDWs in this study also solicit informational assistance from interpersonal networks and media outlets, although they place more trust in migrant unions and the Indonesian Consulate"(Oktavianus and Lin, 2021)
	Mistry, et al. (2021)	"…We noted the importance of social networks among our study participants"(Mistry et al., 2021)
	Knights, et al. (2021)	"They also reported a loss of access to support networks and community organisations during the pandemic, services that previously helped them to access health care and navigate the healthcare system"(Knights et al., 2021)
	AM, et al. (2021)	"Informal social networks and word of mouth in residential areas were also primarily identified as authoritative sources of information on COVID-19" (AM et al., 2021)
**Family and peer influence**	Zlotnick, et al. (2022)	"…reliance on health information from family and friends was negatively associated with obtaining COVID-19 information on the same day it was issued…(Zlotnick et al., 2022)
	Oktavianus and Lin (2021)	"All participants reported that they drew informational assistance from fellow MDWs in Hong Kong, family members in Indonesia, and employers during the health crisis"(Oktavianus and Lin, 2021)
	Lu and Chu (2022)	"We employed two types of acculturation measures: a more traditional one based on host country language and friends, and another focused on perceived distance from both host and home countries which might be a better representation of the bidimensional model of acculturation"(Lu and Chu, 2022)

	Knights, et al. (2021)	"A variety of information sources were described to support decision making, including advice from peers, social media, religious leaders, or information from country of origin"(Knights et al., 2021)
	Healey, et al. (2022)	"One female community member reported that she relied on male relatives (uncles and brother) to gather information about COVID-19, as they were literate in Arabic"(Healey et al., 2022)
	AM, et al. (2021)	"Informal social networks and word of mouth in residential areas were also primarily identified as authoritative sources of information on COVID-19"(AM et al., 2021)
	Liem, et al. (2021)	"In the absence of COVID-19 information provided by official government public health sources in their native language, migrant workers are likely to have relied on informal sources of information, including peer networks and online social media"(Liem et al., 2021)
**Media use**	Zamil, et al. (2022)	"Interestingly, this age group represents the largest age demographic in the study that is represented on the social media site TikTok, as nearly two-thirds of the site's user base is under the age of 29"(Zamil et al., 2022)
	Wang, et al. (2020)	"Our study showed that social media was the main channel of receiving information among international migrants in China regarding COVID-19, which could be a useful platform for providing timely information and other forms of social support during the epidemic"(Wang et al., 2020b)
	Oktavianus and Lin (2021)	"Furthermore, the participants consumed numerous media outlets, including ethnic, local, and homeland media. Many participants shared that they learned about the situation in Hong Kong and Indonesia from the Indonesian ethnic press in Hong Kong, which published news in Indonesian"(Oktavianus and Lin, 2021)
	Narla, et al. (2020)	"Importantly, the study confirmed that these women had access to smartphones and that they use them as a method of accessing health information"(Narla et al., 2020)

	Kiyohara, et al. (2022)	“Our first finding that migrants hardly accessed public multilingual websites and relied on Facebook as a source of COVID-19 information was consistent with previous research”(Kiyohara et al., 2022)
	Harris, et al. (2021)	“Identified channels of information on COVID-19 were via the internet and social media sites including Facebook, Twitter, and Instagram”(Harris, 2021)
	Goldsmith, et al. (2022)	“Among migrant and ethnic minority populations in the UK, US, China, Jordan, Qatar, and Turkey we found evidence of consistent use of social media for COVID-19 information, including via WeChat, Facebook, WhatsApp, Instagram, Twitter, YouTube, which may stem from a difficulty in accessing COVID-19 information in their native languages or from sources they trusted”(Goldsmith et al., 2022)
	Feinberg, et al. (2021)	“Although mistrust and misinformation are not new public health phenomena, the recent proliferation of social media provides a platform where falsehoods are both created and disseminated quickly”(Feinberg et al., 2021)
	Brønholt, et al. (2021)	“Social media thus acted as a key gateway to receive health and risk information about COVID-19 when official information channels were inaccessible”(Brønholt et al., 2021)
	Knights, et al. (2021)	“Additionally, they reported views of COVID-19 and COVID-19 vaccinations that ranged from acceptance to misinformation, often originating from social media or word of mouth.” (Knights et al., 2021)
	Liem et al. (2021)	“In the absence of COVID-19 information provided by official government public health sources in their native language, migrant workers are likely to have relied on informal sources of information, including peer networks and online social media” (Liem et al., 2021)

	Lu and Chu (2022)<br><br><br><br>Hamadneh et al., (2021)	"Relatedly, participants' general perceived information-gathering capacity, which was not framed specifically for a particular type of media, increased their use of both U.S. and Chinese media (Lu and Chu, 2022)<br><br><br>"…For a large part, this could be because of the wide coverage through all types of media" (Hamadneh et al., 2021)
**Physical environment**	Deal, et. al. (2021)	"The worsening of psychosocial stressors, such as fear of eviction; loss or reduction of livelihood; gender-, religion-, or ethnic-based discrimination; sexual and gender-based violence; confinement measures and overloaded residential resources or homelessness; and stigmatization due to mental health issues are main sources of distress in these populations"(Deal et al., 2021)
