# Supplementary figures and images for: Antecedents and Consequences of Health Literacy among Refugees and Migrants during the First Two Years of COVID-19: A Scoping Review
Source: Trop Med Infect Dis. 2024 May 16;9(5):116. doi: 10.3390/tropicalmed9050116 (PMC11126087; doi:10.3390/tropicalmed9050116)

Figure S1 – PRISMA-ScR Flow Diagram (30)

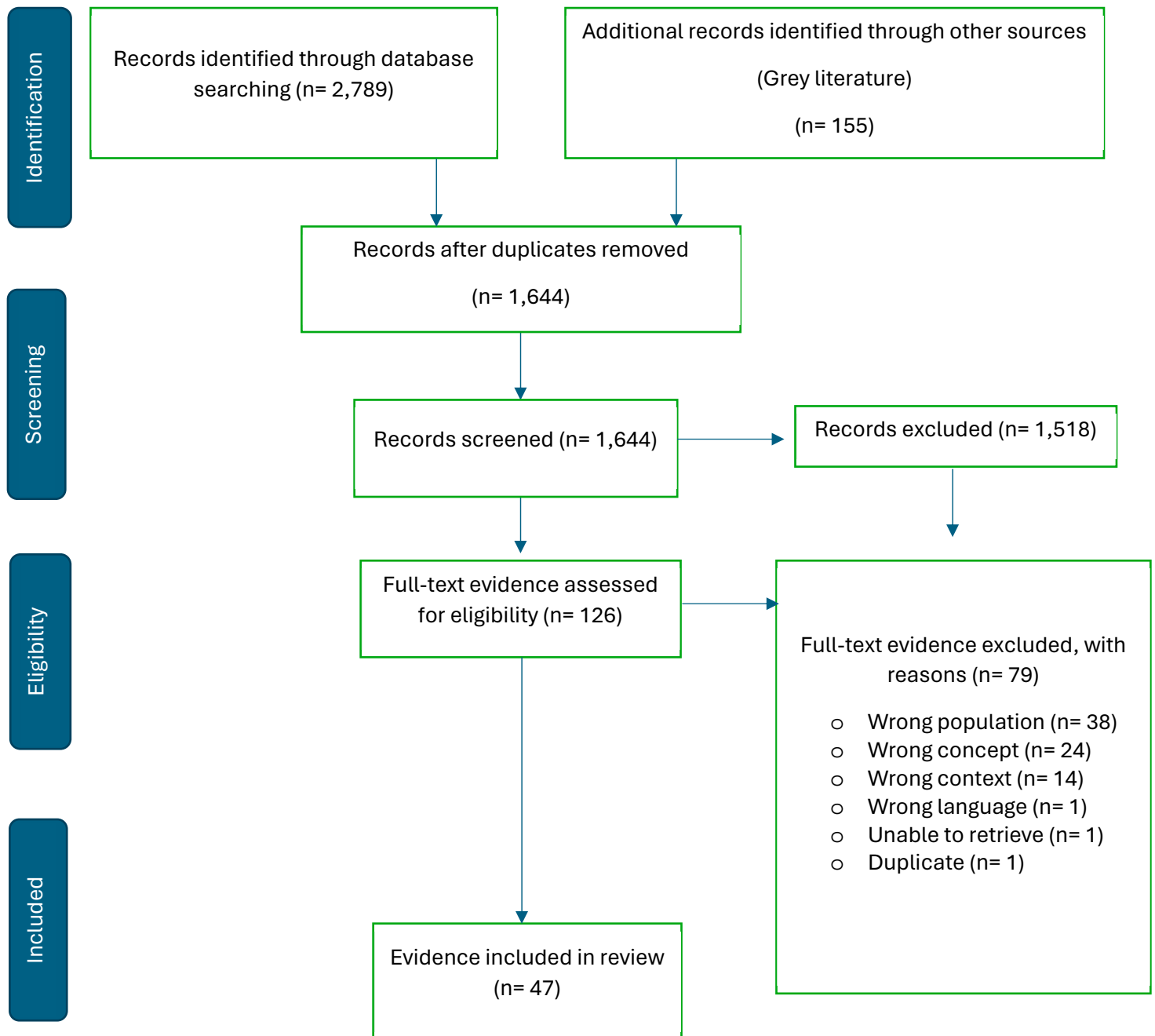

Supplement: Supplementary file 1 [file tropicalmed-09-00116-s001.zip › Supplementary file S6.pdf]
